# Situational Awareness: Mapping Interference Sources in Real-Time Using a Smartphone App

**DOI:** 10.3390/s18124130

**Published:** 2018-11-26

**Authors:** Hong Lam Nguyen, Micaela Troglia Gamba, Emanuela Falletti, Tung Hai Ta

**Affiliations:** 1 Politecnico di Torino, Corso Duca degli Abruzzi 24, 10129 Torino, Italy; honglam.nguyen@studenti.polito.it; 2 Hanoi University of Science and Technology, No. 1 Dai Co Viet, Hanoi 10000, Vietnam; tung.tahai@hust.edu.vn; 3 Istituto Superiore Mario Boella (ISMB), Via P.C. Boggio 61, 10138 Torino, Italy; falletti@ismb.it

**Keywords:** interference detection, Android, GNSS software receivers

## Abstract

In the past years, many techniques have been researched and developed to detect and identify the interference sources of Global Navigation Satellite System (GNSS) signals. In this paper, we utilize a simple and portable application to map interference sources in real-time. The results are promising and show the potential of the crowdsourcing for monitoring and mapping GNSS interference distribution.

## 1. Introduction

Radio-frequency interference (RFI), either unconscious or intentional, is one of the most feared events that can disrupt the functionalities of a Global Navigation Satellite System (GNSS) receiver and the user-level applications dependent on it [1,2]. The importance of creating a ‘situational awareness’ around the receiver, in order to recognize the situation in which an unwanted RFI prevents the correct functioning of the receiver and to react properly without ‘domino’ effects on the application layer has been widely argued [3,4].

Receivers with fully-capable RFI detection modules have been so far quite complex and, translating complexity into costs and size and power consumption, limited to specific professional or military applications [3,5]. On the other hand, the close advent of a multiplicity of payment/finance applications based on GNSS is today considered a fact [5,6], as well as the need for high positioning accuracy and reliability expressed by automated driving applications [7,8]. Contexts like these foresee a massive deployment of ‘consumer-grade’ receiver chipsets, in which not only each on-field receiver needs to be aware of the levels of RFI it is surrounded by to properly react in real-time, but it can be an added value for the application service provider to have a real-time map of the RFI over wide areas, for example to possibly take preventive actions.

Several past works have reported the creation of interference source maps in the GNSS bands, through various data collections performed ad-hoc for testing specific detection, mitigation, or localization algorithms [9,10]. However, those data collection campaigns are unfit for the applications mentioned before, because they are meant to offer a representative sample of the average interference scenario in a certain environment in non-real-time, while they are evidently unable to offer a real-time picture of the RFI nearby a certain position.

In our work we leverage the huge computational capabilities offered today by an octa-core commercial smartphone to run on it an instance of a software GNSS receiver, used as a portable and easily deployable “early stage RFI detector”; we named this software receiver ‘NGeneApp’, as it is the App evolution of our original software receiver for standard PC ‘NGene’ [11,12]. In this way, equipped with a smartphone with an enabled cellular data connection, an external finger-size radio front-end (FE) and a very low cost GNSS antenna, we are able to create in real-time a map of the interference along a certain travelled path. The sensed interference environment is sent to a server machine in our lab acting as a “control center”, where the interference level can be monitored. In perspective, a simultaneous deployment of several such smartphones could allow the creation and continuous monitoring of an interference map over certain areas of interest.

The on-chip receiver of modern smartphones can already provide some measurements via NMEA messages, which can be exploited to detect interferers. For example, a post-correlation technique is used in [13] where a jammer localization is performed using carrier-to-noise power ratio (C/N_0_) measurements provided by Android smartphones. However, the C/N_0_ level may depend on several factors that can impair the clean reception of the GNSS signal, such as shadowing, non-line-of-sight propagation, partial blockage, multipath; furthermore, in case of a strong interfering power the receiver may be completely blinded and may lose signal tracking. On the other hand, since 2017 the top-level Android smartphones have started to provide ‘raw’ GNSS measurements, namely carrier and code measurements, decoded navigation message, as well as Automatic Gain Control (AGC) levels, through an ad-hoc Application Programming Interface (API) [14,15]. This innovation has followed the idea of opening the GNSS signal processing chain before the final on-chip Position-Velocity and Time (PVT) solution, to allow third-party processing capabilities based on non-standard algorithms to improve GNSS performance: for example, aided positioning, differential positioning, precise point positioning. The availability of such measurements, together with the intrinsic network connectivity, can be also exploited to implement forms of distributed interference monitoring, as investigated in [16,17]. The exercise demonstrated with NGeneApp goes further in this direction, exploiting lower-level measurements still not accessible in the commercial chipsets, with the purpose of precisely and flexibly monitor the presence and the impact of interference sources, using an approach that directly enables distributed interference mapping via crowdsourcing of data. 

We organize this work in the following sections: Section 2 discusses the theoretical background; Section 3 is a description of the instrumentation, with particular emphasis to the software receiver ported to the microprocessor of the smartphone; Section 4 describes the RFI detection algorithms implemented on the smartphone; Section 5 describes the validation tests and calibrations performed with lab instruments on the RFI detection module; finally, Section 6 reports the observations from some live measurements around the city. Finally, Section 7 draws the conclusions and discuss the expected developments of the work.

## 2. Theoretical Background

Any RF signal from an undesired source that affects a GNSS receiver is considered interference [18]. According to its source, RFI can be classified as unintentional or intentional. While the former, including out-of-band emissions and harmonic frequencies of commercial systems, is accidental, the latter is specifically generated to deny the GNSS service and, due to its malicious origin, it is certainly the most dangerous one. Among the intentional sources of interference, jamming and spoofing are the two main categories and much effort is being spent by the research community in tackling such kind of threats. Jamming consists in injecting a high power signal into the GNSS band with the final purpose of disturbing/blocking the reception of GNSS signal. Even more malicious than jamming, a spoofing attack deceives a target receiver with a counterfeit copy of the GNSS signal to take control of the receiver behavior. Several recent newspaper and magazine articles report jamming events [19,20,21] and spoofing attacks [22,23]. In this context, the need of creating a ‘situational awareness’ around the receiver is more than ever required [24,25]. In this regard, the crowd-sourcing paradigm, i.e., aggregating and sharing information from multiple receivers, is an highly effective means for detecting and even locating interference sources, as shown in [26] for the detection of spoofing attacks in the aviation domain and in [25] for creating situational awareness for vessels sailing the Baltic Sea.

Many are the approaches presented so far to deal with jamming and spoofing signals. Comprehensive surveys of the main state of art solutions can be found in [27,28] and [29,30,31,32,33,34], respectively. Among all of them, the detection algorithms are of primary interest: in order to trigger any procedure of classification, localization and mitigation, the interference has to be at first detected. In this context a quick crowdsourced detection is recommended to raise early warnings and take preventive actions.

It has already been argued that an effective detection relies on a combination of techniques applied at different stages along the GNSS signal processing chain [2]. In the literature, most solutions propose a mixture of pre- and post-correlation techniques for a single antenna stand-alone GNSS receiver. While the former detects the presence of an unexpected distribution of power in GNSS bands, the latter are used to find an abnormal behavior of correlation peaks. The clear trend is to design algorithms able to detect and classify all kind of interference [34,35,36,37,38,39]. For instance, in [34] a simple and low-complexity power-distortion detector, able to discriminate jamming from spoofing and multipath signals is presented: it is based on observations of both the received power and correlation function distortion, used into a Bayesian M-ary hypothesis testing framework. A combination of pre- and post-correlation metrics is proposed in [35], which employs both an input data variance plus PSD analysis and a C/N_0_ plus symmetric ratio test. Pre–correlation time-frequency (TF) domain analysis, mainly focusing on jamming signals, is adopted in [37,38,39]. Targeting specifically spoofing detection, works in [40,41] propose post–correlation approaches based respectively on a new particle filter for the positioning computation and detections’ fusion based on correlators output monitoring and Doppler consistency check. A subspace projection-based spoofing mitigation algorithm, relying on code delay and Doppler information, is detailed in [42].

A combination of pre- and post-correlation techniques for the RFI detection is also the approach adopted in this work. As better detailed in Section 4, we propose a new simple PSD-based metric, namely the total energy of error, to be applied to the pre-correlation samples and a Signal Quality Monitoring (SQM) algorithm, namely the Chi-square Goodness of Fit (GoF) statistical test [43], to the post-correlation samples. The effectiveness of the GoF test to detect continuous wave (CW) interferers and spoofing attacks was proved in [44,45,46], while its validity with jamming has not been fully investigated yet. In this paper the selected algorithms, included the GoF test, has been analyzed to deal with jamming signals and properly modified to target a real-time Android based implementation, which is the main focus of this work. Although calibrated on jamming interferers, it is worth noticing that the proposed detection methods are not limited to that specific interfering signal. Being able to detect any abnormal received power and correlation distortions, their effectiveness with other kinds of interference cannot be excluded. The goal is to quickly and successfully detect the presence of a disturbing signal, while the classification of such disturbance is left for future work. 

## 3. NGeneApp: An Android-Based Real Time RFI Detector for Smartphones

In order to detect the interference in the surrounding environment in a quick and practical way, we ported a GNSS software receiver to a pocket-size and portable device, i.e., onto the microprocessor of a smartphone. We developed an Android app to run a GNSS software receiver, able to detect in real-time RFI and send the collected data to a remote server. The Android-based real-time GPS/EGNOS/Galileo single frequency fully software (SW) receiver, named NGeneApp, has been obtained porting the source code of an ARM-based SW receiver [12] to the Android Operating System (OS). The smartphone is then connected to a mass-market USB-based FE and a classic hemispherical patch antenna. 

### 3.1. General Overview of the Development Work

Portability, compatibility, and flexibility are the three key drivers for the choice of the target smartphone used to develop NGeneApp. Among all the OSs currently available in the market place, Android OS grants the highest level of flexibility and portability. Since Android is an open platform based on the Linux kernel, developers can even access the file system if they have root permission; furthermore, the massive number of Android devices with several hardware capabilities and features is an advantage in terms of development support and tools stability. On the other side, the hardware compatibility with respect to the original source code is highly desired to ease the porting procedure. From all these considerations and after a scouting the market, to compare different solutions in terms of performance, power consumption and price, the Samsung Galaxy S6 has been selected as target smartphone: it features a 64-bit Exynos 7 Octa 7420 system-on-chip, which consists of a Quad Core 2.1 GHz Cortex-A57 and a Quad Core 1.5 GHz Cortex-A53. The choice of the ARM family as processor architecture allows the full portability of the Single-Instruction Multiple-Data (SIMD) NEON instructions exploited by the original source code to satisfy real-time requirements. The entire development work was performed on a laptop PC, running Windows 10 OS, using Android Studio, which is the official Integrated Development Environment (IDE) for building app on Android devices. 

Although based on the Linux kernel, Android OS shows one main difference compared to most of desktop Linux distributions: the default factory configuration does not grant root access to the OS. NGeneApp, on the contrary, requires administrator privilege in order to read raw samples coming from the USB-based FE through libusb library functions calls. Thus, the ‘rooting’ procedure was the first necessary step in the development chain. The second step of the porting procedure was the compilation of the original code on the target platform: the native code can be called in Android application through the Java Native Interface (JNI); Android Studio provides the Native Development Kit (NDK) toolset to compile C and C++ code into a native library and packs it into an Android Package Kit (APK) using Gradle, the IDE’s integrated build system. After the resolution of some libusb compatibility issues, the porting of the whole native code (ANSI-C and assembly) was accomplished. At this point, the two functionalities that distinguish NGeneApp have been implemented in the form of two additional software modules: the remote server communication and the RFI detection. The communication between NGeneApp and the server is established by using the TCP/IP protocol via network sockets. NGeneApp acts as a client, sending the request to the server every five seconds until the connection is established. After the communication initialization, NGeneApp sends a data message to the control server every second. The connection with the server, which is actually a PC located at ISMB premises, is set up through the smartphone data uplink, exploiting both Wi-Fi and the cellular network, i.e., mainly 4G Global System for Mobile Communication (GSM). The detailed description of the adopted RFI algorithms, coded in ANSI-C, is deferred to Section 4. The next two sections describe respectively the high-level architecture of NGeneApp including the list of main functionalities, and the smartphone-dependent optimizations implemented to fully exploit the hardware resources.

### 3.2. NGeneApp High-Level Architecture

**Figure 1:**
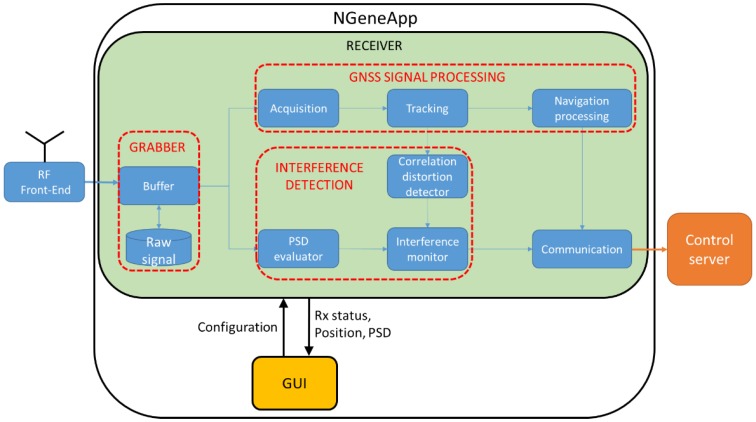
NGeneApp high-level architecture.

As shown in Figure 1 the high-level architecture of NGeneApp consists of two main blocks: the Graphical User Interface (GUI) and the receiver. The GUI, written in Java, has been designed to allow the user to set the configurable parameters, to interact with the receiver, and to display some basic information such as the receiver status and the tracked satellites. Then, like any other satellite navigation tool, the real-time position computed by receiver can be shown in Google Maps thanks to the Google Map API in Android. In addition to these basic functionalities, the GUI is able to plot the Power-Spectral-Density (PSD) of the raw samples coming from the FE, by using a charting library called Achartegine [47], so that the user can monitor the presence of interference in real-time. 

The NGeneApp’s receiver includes four modules, also illustrated in Figure 1:

- the *grabber*, which consists of a function that stores the raw GNSS samples coming from the FE to the internal memory of the smartphone, for post-processing analysis;
- the whole GNSS signal processing chain, from acquisition to PVT computation, for the real-time processing of the raw GNSS samples coming from the FE;
- the interference detection functionality, working in real-time and implemented at two stages, as better detailed in Section 4:
  ○ Early stage detection, by means of a spectral analysis, called PSD evaluator in Figure 1;
  ○ Intermediate stage detection, by means of a correlation distortion monitoring technique, as shown in Figure 1;
- real-time server communication and data storage for interference distribution monitoring in a crowdsourcing perspective: NGeneApp sends a data message containing the receiver measurements to the server every second. The data are processed and stored in the database for mapping and investigating the distribution of interference in the area in real-time. In case the communication connection is lost, the data message is kept in the local memory of the device and will be resubmitted to the server right after the network is available again.

NGeneApp can be executed in two modes:

1. Grabbing mode: NGeneApp stores the raw GNSS samples coming from the FE to the internal memory of the smartphone. In this mode, only the *grabber* module, as depicted in Figure 1, is enabled.
2. Receiver&RFI detector mode: NGeneApp acts as a complete GNSS receiver and enables its capabilities of RFI detection and transmission of data to a remote server. The user can further specify the data source and its associated processing mode:
  - *Real-time*: the raw GNSS samples come at high rate (tens of MHz) from a USB-based FE and are processed on the fly;
  - *Post-processing*: NGeneApp reads the GNSS data from a file. In this case, no real-time requirements have to be satisfied.

Table 1 reports the main features of the FEs currently supported by NGeneApp, in terms of sampling frequency, intermediate frequency (IF) and FE bandwidth. It is worth noting that, thanks to the Software Defined Radio (SDR) approach, NGeneApp can be used with other FE parameters combinations, allowing the user to specify its own tailored setup, taking into account that the real-time requirements have to be satisfied. The sampling frequency is upper limited by the USB transfer speed and the hardware capabilities. Both the SiGE v3 [48] and the STEREO FE [49] are configurable, thus the configuration reported in the second and third rows of Table 1 represents just one of the many possibilities. Other FEs are currently under evaluation. For the purpose of a portable and easily deployable RFI detector, an additional aspect that cannot be neglected is the power supply mode required by the FEs. Both SiGE v2 and v3 can be powered by the smartphone USB, so both are suitable to be used for on-field tests. The STEREO FE, on the contrary, needs an external power supply, to be provided via an additional portable charger.

### 3.3. The Crowdsourcing Approach of the Server

For saving bandwidth and further analysis in case of interference, the information sent to server is classified into two types of message. The first type of message, which is regularly sent to server, contains the following information:

- The PVT results computed by NGeneApp receiver
- The PSD estimation and the total energy of error value
- The correlation distribution of the Chi-square GoF test

The second type of message will be sent when the interference is detected consist of the following information:

- 30 s of IF digitalized samples (raw data)
- The output of the tracking stage (i.e., correlators value, Doppler frequency, code rate)
- C/N_0_ measurements

By using the PVT results and the C/N_0_ from the crowd-sourced, the possibility of detecting and localizing the source of the jammer was demonstrated in [17,51]. In those studies, the information (i.e., C/N_0_) is extracted from the GNSS receiver embedded in smartphone. However, the C/N_0_ value may be affected by other factors, such as multipath and partial blockage. Therefore, when the interference is detected, NGeneApp sends also the spectrum and raw data which enable the server to identify the source of jamming in a more accurate and flexible way [9]. An analysis of the advantages and drawbacks of the proposed crowdsourcing approach versus other similar works is reported in Table 2.

The proposed server can also characterize the interference by using the spectrum data. Moreover, the raw data sent simultaneous from multi-source is also valuable for further investigation. With this IF samples database, the interference event can be analyzed and replicated in post-processing investigation. Hence, we can evaluate the effect of interference on the receiver operation and assess the performance of receiver under harsh environment [54,55].

### 3.4. Hardware-Dependent Optimizations

**Figure 2:**
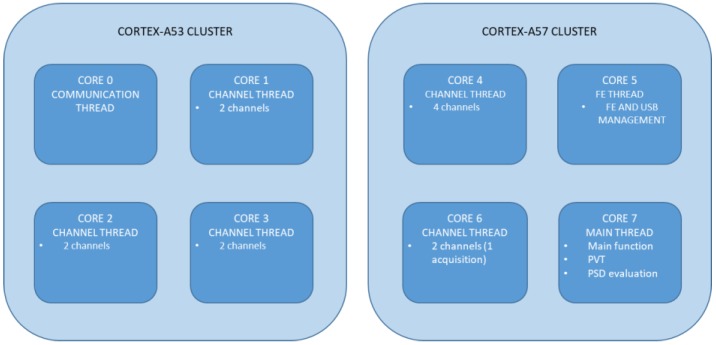
Thread allocation of NGeneApp on the cores of the Samsung Galaxy S6.

In order to fulfill the real-time requirement, some smartphone-dependent optimizations are required. The interference detection functionality demands a very high computational burden, thus, the multi-thread programming needed a threads re-distribution among all available processor cores, to fully exploit the benefit of the high performance chipset. Figure 2 represents the threads allocation onto the eight cores of the Exynos 7 Octa 7420 system-on-chip. 

Being specifically designed for high performance applications, the Cortex-A57 cluster is in charge of handling all the threads with high priority or computational burden. For example, the FE thread, in charge of handling all functions related to the USB FE and stream, is allocated to one core due to the high data rate. The main thread including the PVT computation, the PSD estimation and the ‘main’ receiver function is mapped to the core no. 7 while a channel thread is assigned to each of the two remaining cores in the cluster. The channel thread includes all the operations needed to track a certain number of satellite signals and to perform the intermediate stage interference detection. Thus, cores no. 4 and 6 can manage up to six channels, i.e., six satellites in tracking. Signal acquisition, which is the heaviest function in terms of computational burden, is continuously performed on one channel on core no. 6, until all the channels are in tracking state. Being optimized for power efficiency, the Cortex-A53 cluster is less powerful compared to the Cortex-A57 also in terms of clock frequency (1.5 GHz vs. 2.1 GHz). Thus, in this case, three cores handle two channels each one. The remaining core executes the communication thread, in charge of handling the remote server link, which requires high priority for the timely delivery of the messages. Taking advantage of this threads allocation, NGeneApp is currently able to handle up to 12 channels in real-time.

## 4. In-Field Interference Detection Modules

According to the theoretical background in Section 2, the best way to detect the interference is to monitor its effects along the receiving chain. For this reason, the detection module of NGeneApp includes so far two monitoring points: the first one on the pre-correlation samples, based on PSD evaluations, the second one on the post-correlation samples, based on the Chi-square GoF statistical test [43]. The two techniques are briefly described hereafter.

### 4.1. Power Spectral Density (PSD) Monitoring

A well-known pre-correlation technique consists in monitoring the digitalized samples at the FE output by means of a PSD evaluation. In this case, any interferer with power level exceeding the noise floor can be detected by comparing the PSD of the incoming signal with a pre-set threshold mask. This method can detect the appearance of a disturbance at a very early stage, warning the user in real-time, and this is the approach we use in NGeneApp. Together with a post-correlation technique to assess the actual impairment to the receiver operations, this method has the potential to provide a reliable real-time interference detection tool. This technique works on the raw samples produced by the digital FE, therefore NGeneApp is the suitable tool to access those samples through the USB connection with the FE; the same processing cannot be directly applied to the commercial chipsets, because they do not provide the intermediate frequency signal samples. In the crowd-sourcing perspective, the mobile network can be exploited to send detected interference information to a remote server, for mapping, monitoring and analysis purposes.

The spectral estimation method implemented in NGeneApp is the normalized Welch periodogram, based on the average of a sequence of windowed Fast Fourier Transforms (FFTs) computed over 4096 points. A threshold mask mechanism is currently applied, to detect and roughly classify GNSS interference source, either CW or wideband. A calibration phase has been performed in laboratory in order to properly set the detection masks as a function of the detectable Interference-over-Signal power ratio (I/S). In fact, the threshold was set based on the *total energy of error (TE)* between the computed PSD and the PSD evaluated in the interference-free environment. The TE was computed as:

$$TE=\overset{N}{\underset{i=1}{\sum }}{\left(C_{i}-R_{i}\right)}^{2}$$

where N is number of frequency samples per spectrum, $C_{i}$ and $R_{i}$ are the PSD values computed at frequency point i of the current PSD and the reference PSD, respectively.

When no interference is detected (TE under threshold), the computed PSDs are sent to the server with a rate of 1 Hz, while when a disturbance is noticed (TE above threshold), the transmission rate is increased to 5 Hz (selectable).

### 4.2. Chi-Square Goodness of Fit (GOF) Test

The second interference detection technique implemented in NGeneApp is the Chi-square GoF test, which acts a post-correlation monitoring point along the receiver chain [43]. It is based on a test statistic for monitoring the distortion of the signal correlation function in the tracking stage of each received satellite signal. 

The algorithm is based on the fact that in the nominal case, i.e., when no interference is present, the code correlation for each satellite signal is an even function; each pair of Early (E) and Late (L) correlators equally spaced from the Prompt (P) (the early-prompt spacing and the late-prompt spacing are equal, i.e., $d_{EP}=d_{LP}$) can be modeled as a pair of normally distributed random variables with the same mean, i.e., $\mu_{E}=\mu_{L}=\mu$, and variance that depends on the (C/N_0_). On the other hand, in the presence of an interfering signal, the E and L point correlation distributions significantly differ, because of the induced code correlation distortion. If the early-late spacing $d_{EP}$ exceeds 1 chip, then it is possible to show that E and L are independent and D=E−L results to be a normally distributed random variable with zero mean, $\mu_{D}=0$. Then, the test statistic is built on the vector of differences D. Based on these assumptions, the GoF algorithm consists in evaluating the distribution of D, against the expected distribution, i.e., the one calibrated in nominal conditions. The GoF is able to estimate how much the two distributions differ, by means of a statistical metric, the so-called *p*-value, which is the probability that the two distributions have the same statistical characteristics. When no disturbances affect the signals and the correlation shape is not distorted, the distribution of D is similar to the one calibrated in nominal conditions, and the *p*-value is close to one. On the contrary, in a critical scenario where interference distorts the correlation, the *p*-value assumes smaller values. A threshold mechanism is used to decide on the binary hypothesis, set on the basis of the ‘significance level’ of the test [44]. For a thorough theoretical description of the GoF statistical test, the reader can refer to [43,44]. Hereafter, the in-laboratory calibration phase as a function of the C/N_0_ signal ratio in nominal conditions and the on-field test results are presented in Section 5.2 and Section 6.3 respectively.

## 5. In-Laboratory Tests and Calibrations

**Figure 3:**
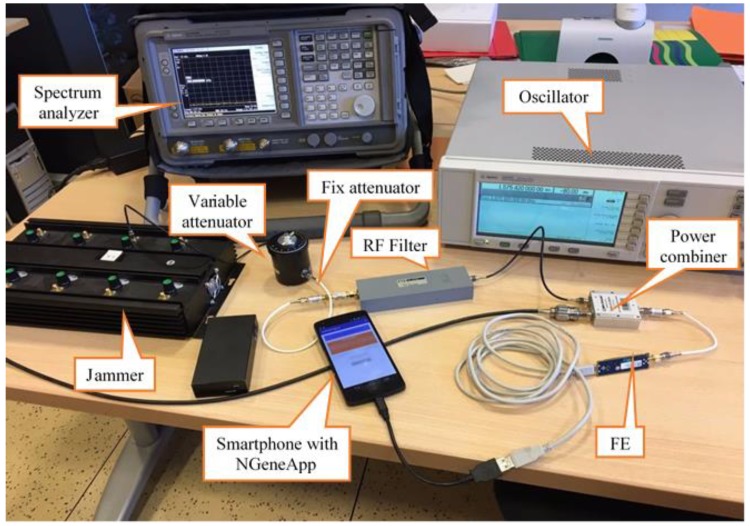
In-laboratory test setup.

The capability of NGeneApp of acting as an interference detector was first assessed with in-laboratory tests, aimed at determining the detection sensitivity of the App to two basic kinds of interference: wideband noise and CW. This test campaign also served to calibrate the spectral detection masks and GoF reference correlations. A picture of the experiment setup is shown in Figure 3.

### 5.1. Spectral Detection with Wideband Interference

**Figure 4:**
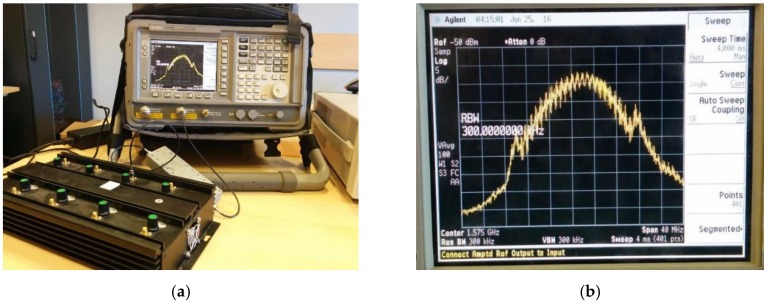
Spectral analysis of the jammer on the L1 band (**a**) and zoomed-view (**b**).

In the first experiment a wideband jammer, visible in Figure 3, was employed. It features eight RF outputs, covering different frequencies, including the GNSS L1 band. The power of the generated wideband noise, measured over the GNSS FE bandwidth with a spectrum analyzer connected via RF cable, was −60 dBm (Figure 4).

The jamming source was wired to attenuators in order to control its power with respect to the GNSS signal. Two kinds of attenuator were used: a variable attenuator (0 ÷ 20 dB) with 1 dB resolution and two fix attenuators (10 dB and 20 dB). Once attenuated, the jamming signal was combined with the GNSS signal coming from a rooftop antenna. The mixed signal was then sent to the SiGE v3 FE [48], which outputs a 16.368 MHz digitalized signal modulated at intermediate frequency of 4.092 MHz, as indicated in Table 1. This sample stream is processed in real-time by NGeneApp.

We started the test with maximum jamming power, then we decreased the power with −5 dB step using the attenuators, to determine the minimum in-band interference power level whose effect is non-negligible. 

**Figure 5:**
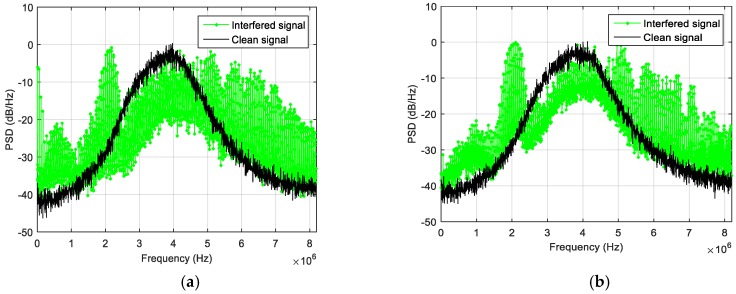

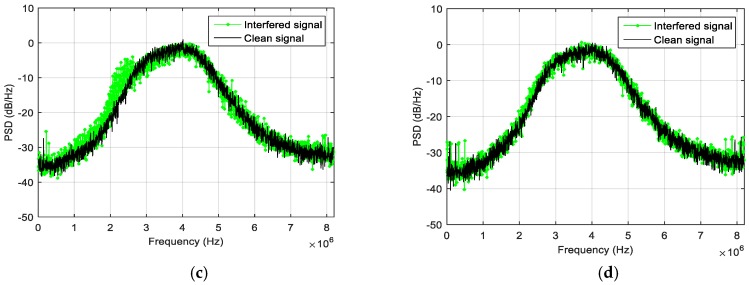
Effect of the wideband jammer on the received PSD for different power levels: −60 dBm (**a**), −80 dBm (**b**), −95 dBm (**c**) and −105 dBm (**d**).

The effect of the jammer has been evaluated both on the PSD of the received signal and on the receiver capability of acquiring, tracking and computing the PVT. Figure 5 summarizes the obtained results in terms of PSD estimation for different level of the interfering power: the green plot represents the obtained PSD when the jammer is on, while the black one is the interference-free PSD. When the jammer power is not attenuated (Figure 5a), the PSD is totally distorted, and the spectral distortion increases dramatically in the whole bandwidth; in this case, the GNSS signal is disrupted and the receiver cannot operate. Till −80 dBm (Figure 5b), the jamming signal has a relevant effect to the receiver performance: when the jammer is turned on, the tracking of some satellite is lost, while some others experience a drop in the C/N_0_ level. No tracking anomaly happens when the jamming level is lower than −85 dBm, but the application is still able to detect the distortion of the spectrum if the interference power is greater than −95 dBm (Figure 5c) where a small distortion in the left side of the spectrum is still visible. Only below −105 dBm (Figure 5d), no distortion is detectable. Based on these observations, the detection threshold for the TE metric was set to 50,000 units. These test results are summarized in Table 3.

### 5.2. GoF Test: Calibration of the Nominal Distributions

The third in-laboratory test aimed to calibrate the reference distribution function of the Chi-square GoF Test. In a previous work [44,45], the reference distribution function was computed and stored during the calibration phase executed in a portion of “clean” signal; in this way, the detection method was calibrated every time a channel starts tracking. The approach [44,45] shows a drawback in the real-time application because it implies repeating the calibration for each channel and for each time the detector starts monitoring, using a portion of non-interfered signal. Therefore, in NGeneApp the calibration phase was performed in the laboratory and the reference distributions are loaded from static memory every time the receiver is switched on. Since the reference distribution depends on the C/N_0_ of the tracked signal, the GoF method implemented in NGeneApp employs the same reference distribution for all the tracking channels which run with similar C/N_0_. 

**Figure 6:**
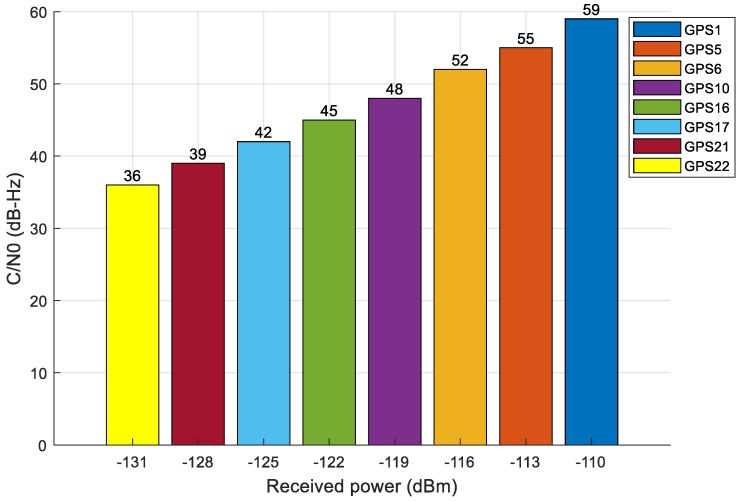
C/N_0_ measurement of the GPS signals used for the calibration of the GoF method.

To compute such reference distributions, we simulated a dataset with eight GPS L1 signals at different power levels, using the NAVX-NCS GNSS signal generator [56]. The received power assigned to the list of satellite signals varied from −110 dBm to −131 dBm, with a step of 3 dB between each pair of received signals. Figure 6 shows the estimated C/N_0_ of each PRN associated to the input power level. From the dataset, the reference distribution function for the Chi-square GoF test of each C/N_0_ level (i.e., PRN) was computed. Then, each reference distribution was used to execute the GoF test on all the simulated signals, to empirically estimate the false alarm rate, which is expected to be zero because the signals are not spoofed. The test was conducted on about 1400 test samples for each signal. The rationale is that, if the reference distribution for a certain input power level keeps the false alarm rate close to zero, then it is suitable for the C/N_0_ of the signal under test. The results of such a calibration test are shown in Table 4. 

We can see that all the PRNs which have C/N_0_ in the same range can use the same reference distribution without significant effect on the algorithm. For example, signals in the range [48,49,50,51,52,53,54,55,56,57,58,59] dBHz (e.g., PRN 1, PRN 5, PRN 6 and PRN 10) could use the reference distribution of PRN 5 with limited false alarm rate. However, from the table, we can also realize that the lower the C/N_0_ value, the higher the false alarm rate. It means that signals with lower C/N_0_ ratio are more sensitive to the change of reference distribution. For example, the GoF test makes the true decision (i.e., authentic signal) for PRN 22 (which has C/N_0_ about 36 dBHz) only when using the exact reference distribution for C/N_0_ = 36 dBHz. In the end, the grouping of C/N_0_ levels that can share the same reference distribution is summarized in Table 5. The same procedure was performed for Galileo E1 signals, obtaining similar results.

## 6. On-Field Measurement Campaigns

**Figure 7:**
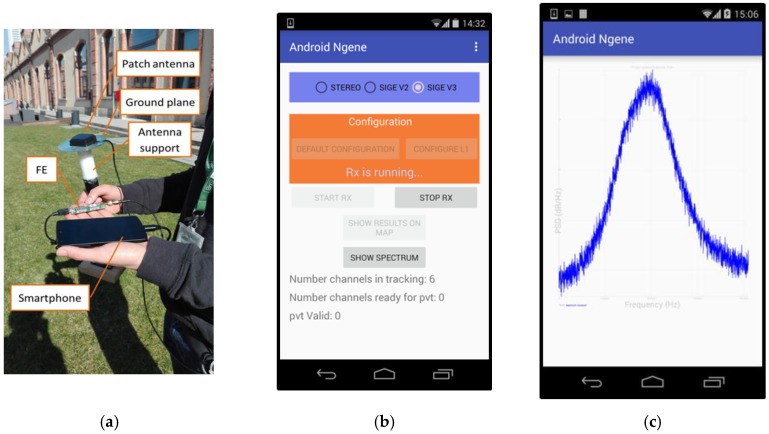
Live experiment test setup (**a**), screenshot of NGeneApp GUI (**b**) and real-time PSD plot (**c**).

A first live test campaign was conducted in order to assess the capability of NGeneApp to catch RF disturbances in real-time and real-life environment. Figure 7a shows the test setup: a smartphone equipped with NGeneApp, a pocket-size FE and a portable patch antenna. We started to map the RF interferences, walking along the streets of the center of Turin, Italy, using this small and lightweight portable equipment. The PSD estimates are displayed on the smartphone and sent to the remote server with a rate of 1 H, increased to 5 Hz when a disturbance is noticed. The GUI and a sample spectrum of received signal are represented in Figure 7b,c, respectively.

### 6.1. Interferences in an Urban Scenario

Three examples of non-harmful interference detected during real-life urban situations are reported in the following case-studies.

#### 6.1.1. Case-study A: Experiment Performed in Porta Nuova Train Station

**Figure 8:**
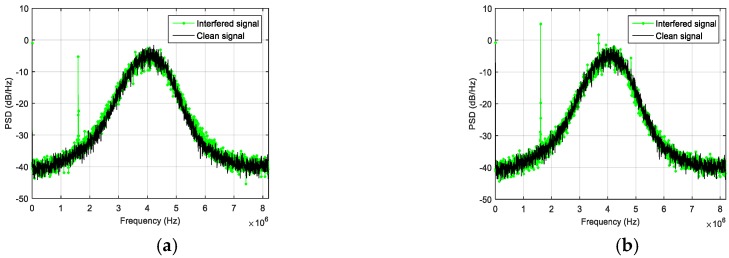

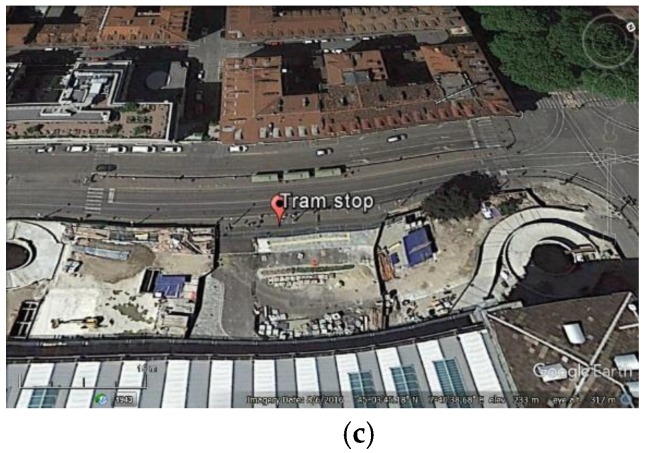
Case-study A. Narrow-band interferer detected on 6 March 2017 at 15 h:26 m:11 s (**a**) and 15 h:28 m:31 s (**b**) local time, at the bus stop n. 253 (Porta Nuova) in Turin (**c**).

Figure 8 shows a disturbance detected on 6 March 2017 in front of Porta Nuova train station in correspondence of the tram stop (Figure 8b). The interesting phenomenon observed is the occurrence of a very narrow-band interference each time a bus or a tram crosses the bus stop (Figure 8c). Figure 8a,b report just two of a series of recorded PSDs, where the peak always appears in the same frequency range: (1.59–1.61) MHz. This anomaly was observed for all the buses and trams passing by the stop. The TE in such cases was 12,000 (dB/Hz)^2^, which was under threshold because the interference in this case have very narrow band and it did not affect considerably the TE value. 

#### 6.1.2. Case-Study B: Experiment Performed on the Road along Corso Eusebio Giambone and Corso Cosenza

**Figure 9:**
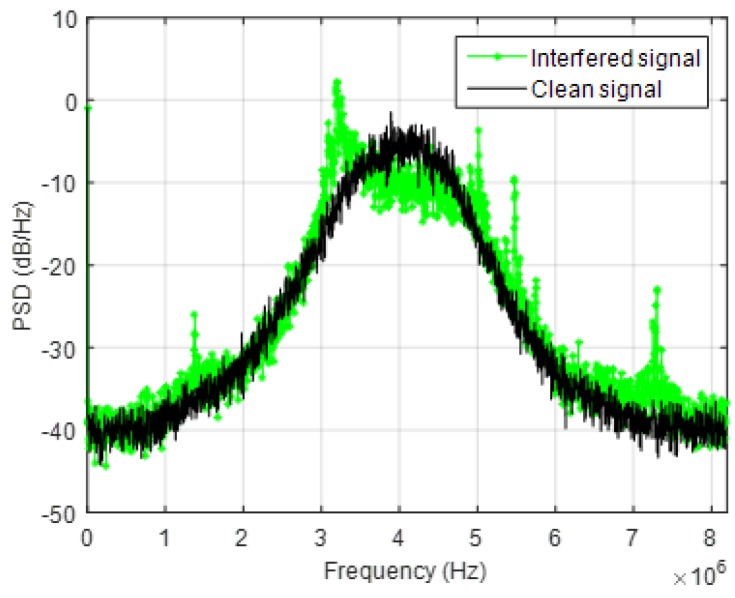
Case-study B. PSD anomaly detected on 7 March 2017 at 11 h:19 m:59 s local time, in Corso Eusebio Giambone, Turin.

Another interesting anomaly was recorded on 7 March 2017, at a specific place in Corso Eusebio Giambone. The evident spectrum distortion is illustrated in Figure 9. This event was noticed each time the receiver passes a pharmacy in Corso Eusebio Giambone, 19. The measured TE was 17,056 (dB/Hz)^2^, which is still under the TE threshold.

#### 6.1.3. Case-Study C: Experiment Performed around Porta Susa Area

**Figure 10:**
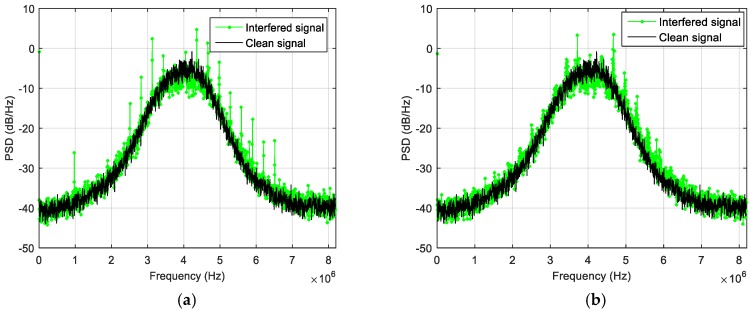
Case-study C. PSD anomaly detected on 7 March 2017 at 12 h:29 m:04 s (**a**) 15 h:30 m:51 s (**b**) local time, in Via Paolo Borsellino, Turin.

During the 7 March test, other disturbances were collected in two places close to the Porta Susa train station. For example, a PSD anomaly has been observed in Via Paolo Borsellino and reported in Figure 10 in two different moments of the day. In this case, the spectrum shows unexpected spikes, however, the anomaly in this place is not persistent.

Apart from the location and address, in all the considered cases it was not possible to locate the interference sources in a more precise way. Anyway, no harmful effect on the receiver operations has been noticed for all the observed disturbances in this scenario.

### 6.2. Detection of an Interference from the Space

On 17 May 2017, a CW interference on the L1 spectrum was detected by the researchers of the NavSAS group, analyzing the signal received from the ISMB rooftop antenna. Two spikes appeared at approximately ±0.5 MHz from the L1 carrier frequency. The phenomenon happened during the afternoon (from about 1.00 p.m. UTC to 6.30 p.m. UTC) and repeated along consecutive days. To investigate the source of this interference, NGeneApp was used as portable RFI detector for dynamic observations (by foot and by car) around the ISMB premises as well as in some other areas of the city, far from the Institute. The spectra observed in all visited areas were similar, with the two spikes always appearing at the same frequency. This fact suggested the intuition that the interference was not a local effect, but something farther, probably originated in space.

**Figure 11:**
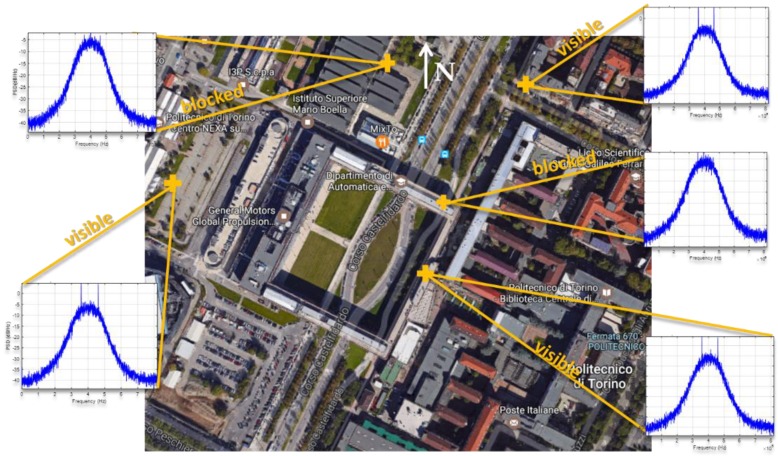
The spectrum observed in the dynamic test.

**Figure 12:**
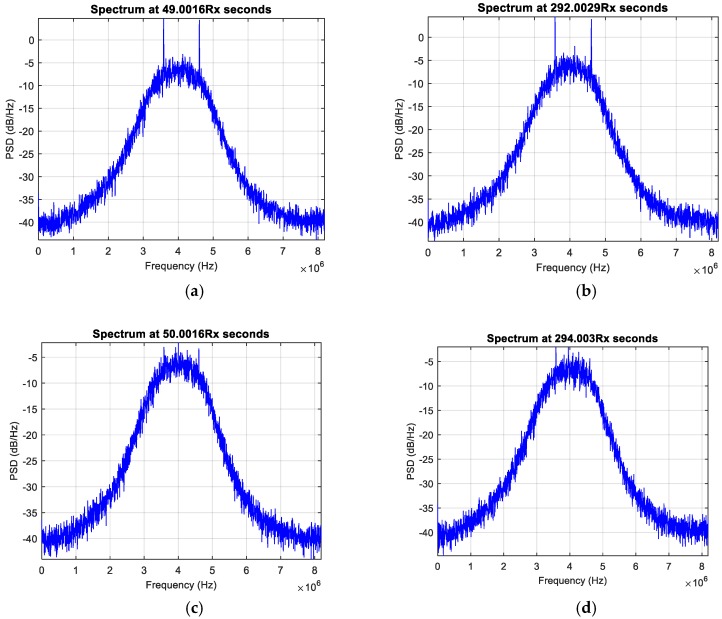
The PSD observed in the afternoon of 19 May 2017: in open sky (**a**,**b**) and when the Western part of the sky was obscured by buildings (**c**,**d**).

**Figure 13:**
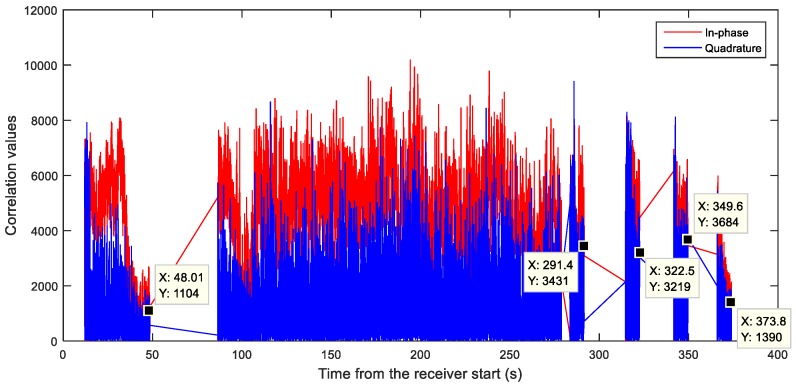
The tracking correlation output of SVN 71 (PRN 26).

Furthermore, during the dynamic observations, when the receiver was moving around a building, the interference seemed to disappear each time the western part of the sky was blocked by the building, as illustrated in Figure 11 and better detailed in Figure 12. Considering the visible duration of the GPS satellites and the direction in the sky, the GPS SVN 71 (PRN 26) was first identified as the potential source of the interference. A dataset was then collected with NGeneApp for the post-processing. Figure 13 shows the correlation output of the GPS satellite SVN 71 performed by the NGeneApp. We can recognize that when the PRN 26 loses the tracking (see cursors info in Figure 13), the two spikes in the spectrum disappeared, as shown in Figure 12a,b at about 50 s and 294 s from the receiver start, respectively.

However, the appearance of the interference did not perfectly match with the visibility of the SVN 71. This mismatch was then explained by the fact that the interfering signal did not come from the SVN 71, but from the non-operational GPS satellite SVN 49 which had a similar orbit to SVN 71. For more details about the analysis of the anomalous GPS signals reported from SVN 49, the interested reader can refer to [57,58].

### 6.3. Interferences from a Complex System

**Figure 14:**
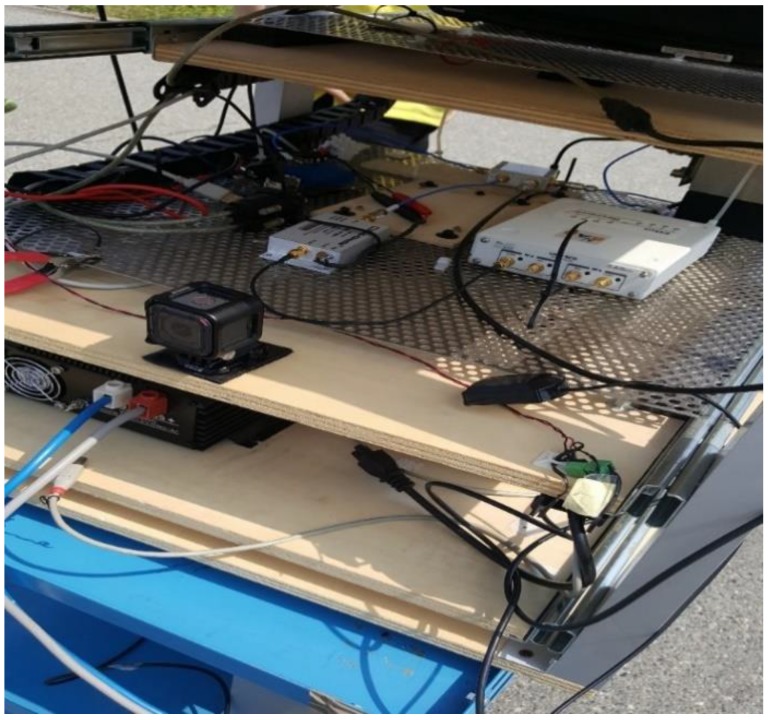
The complex integration setup.

**Figure 15:**
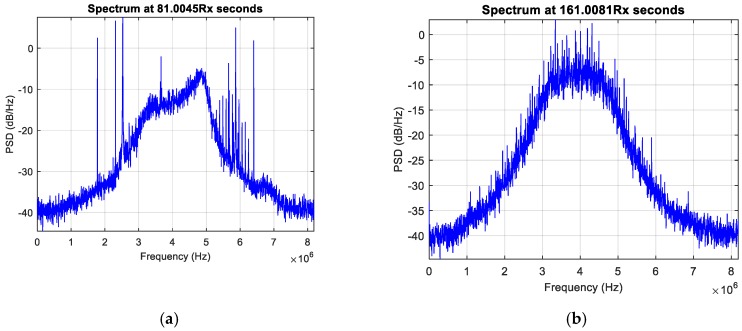

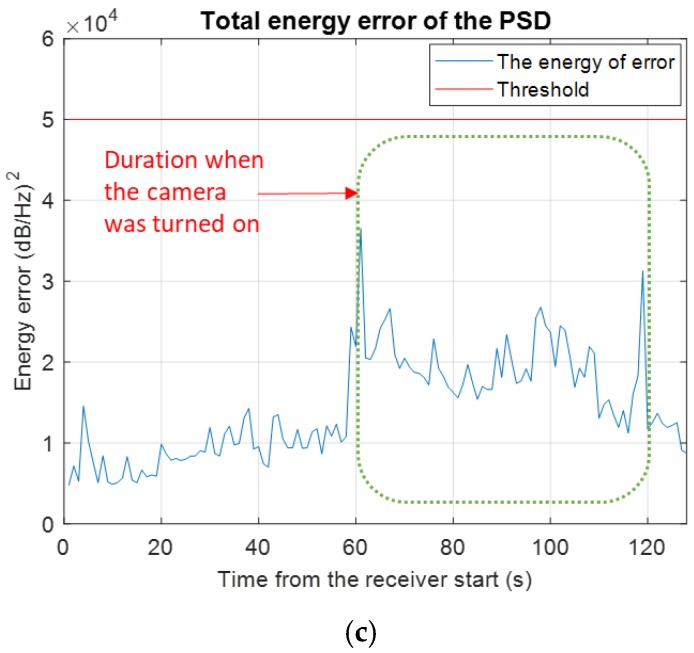
The PSD observed when the camera is (**a**) on (from second 60 to second 120), (**b**) off (the rest) and (**c**) the total energy of error measured during the experiment.

Another anomaly detected and analyzed thanks to NGeneApp is the interference in a complex integration system. The setup included a GNSS receiver, PCs, a GoPro camera, a Universal Software Radio Peripheral (USRP) and a rubidium clock. A photo taken during one of the performed data collections is shown in Figure 14, where the complex integration setup, including the GoPro camera and the USRP, is visible. During the experimental campaign, the researchers of NavSAS group observed some narrow-band interferences on the raw digital samples collected from the USRP. With the spectrum displaying in NGeneApp in real-time, the source of the interference was easily recognized by turning off each device at a time in the system. Finally, the camera was identified as the source of the narrow-band interference. Figure 15a shows the impact of the interference on the spectrum when the camera was turned on. When the GNSS antenna faced the camera at close range (about 10 cm) the spectrum was considerably distorted. After turning off the camera, the shape of spectrum became normal (Figure 15b).

**Figure 16:**
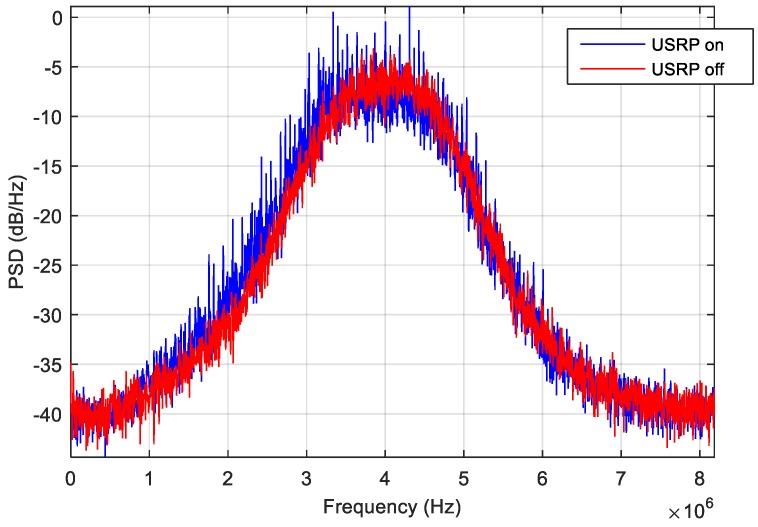
PSD evaluation when the USRP is turned on/off.

**Figure 17:**
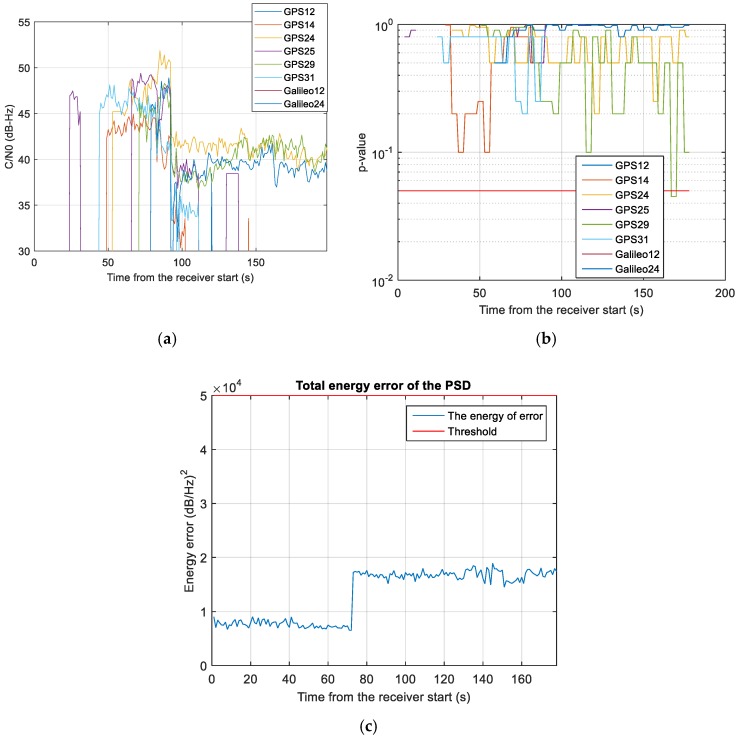
Analysis of the anomaly from the USRP: (**a**) C/N_0_ and (**b**) GoF *p*-values estimated for different PRNs, and (**c**) total energy error of the PSD.

In addition, another anomaly was noticed. In this regard, Figure 16 represents the PSD estimated by NGeneApp in case the USRP was triggered on (blue line) and off (red line). It is evident that although the shape of the spectrum is not distorted, the PSD looks noisier when the USRP is enabled. The effect can be clearly seen in Figure 17a: there is a sudden drop in the C/N_0_ values of about 10 dB-Hz when the USRP starts recording data at about 95 s from the receiver start. However, the interference detector did not raise any warning. In fact, the test metrics produced by the GoF test were always above the detection threshold (i.e., no interference detection, see Figure 17b) meaning that such kind of anomaly did not produce any relevant distortion neither on the correlation function nor on the spectrum. Furthermore, Figure 17c shows a sudden rise in the energy error of the PSD when the anomaly occurs but this increase is not sufficient to trigger the warning. However, when the disturbance occurred, the receiver was significantly affected, so that some channel lose track, for example, GPS PRN14, GPS PRN 25, GPS PRN 31. A possible explanation of this effect is a powerful uniform wideband noise generated by the USRP, which increases the noise floor of the received signal without distorting it. It is clear that, in order to cope with such kind of anomalies, the detection algorithms have to be complemented with additional monitors. For more details about the integration results of this experimental campaign, the interested reader can refer to [59].

## 7. Conclusions and Expected Developments 

In this paper, a new portable and easily deployable real-time RFI detector named NGeneApp has been presented. Particular emphasis has been dedicated to the development work, i.e., to the porting of a software receiver to an Android-based smartphone. The RFI detection functionality has been implemented by means of a combination of pre- and post-correlation techniques, properly calibrated with lab instruments. Furthermore, its effectiveness in catching RF disturbances in real-time and real-life environment has been demonstrated with a live test campaign. In this regard, three main usage examples have been presented and in all of the considered situations, NGeneApp was shown to be able to detect interferences successfully. For instance, narrow band disturbing signals and unexpected spikes, likely unintentional, have been noticed walking in an urban scenario. Using NGeneApp for dynamic tests, the source of an interfering signal coming from space has been quickly identified in a non-operational GPS satellite. Finally, NGeneApp has been employed to quickly recognize potential sources of interference in complex integration systems.

According to the achieved results, NGeneApp shows to be a simple and portable tool to check the presence of interference in the environment in real-time. It has been developed with the potential of being an *in-field sensor* in a de-centralized, unstructured, interference monitoring network. In this network, several sensors spread across wide areas should monitor local GNSS interference, then transmit to a remote server their measurements collected whenever an interference even is detected. Following a crowdsourcing philosophy, a short-delay post-processing implemented at the server side on the data received from sensors would allow drawing a near-real-time map of the interference over a certain area, in order to create conditions of situational awareness. Longer time observations would allow inferring about interference persistence or periodicity and source localization. On the sensor side, the software approach easily enables the potential of enhancing the sensitivity and accuracy of the detection module, for example implementing other detection metrics or refining the detection rules. 

The current version of NGeneApp is able to handle up to maximum 12 channels, which satisfies the interference monitoring requirements. The possibility of using other wider-bandwidth front-ends is currently under investigation, in order to improve the capability of classification and identification of the interference sources. The development of the backend server with monitoring capabilities is another future investigation direction.

## Figures and Tables

**Table 1:** FEs supported by NGeneApp.

Front-End	Sampling Frequency (MHz)	Intermediate Frequency (MHz)	Bandwidth (MHz)
SiGE v2 [50]	16.3676	4.1304	2.5
SiGE v3 [48]	16.368	4.092	2.5
NSL STEREO [49]	16.0	3.905	4.2

**Table 2:** Comparison between similar works and the proposed crowdsourcing approach.

	Other Works [17,51]	Proposed Crowdsourcing
Device	Using embedded GNSS chipset in Android smartphones	❖ Android smartphones
		❖ Front-end (SiGE v2/SiGE v3)
		❖ Patch antenna
Data provided	GNSS information available from the Android OS:	All available information can be gathered from the GNSS receiver:
	PVT results	❖ PVT results
	Satellite information	❖ Satellite information
	C/N_0_	❖ C/N_0_
	Raw measurements such as pseudorange, Doppler frequency, code rate (if supported)	❖ Raw measurements such as pseudorange, Doppler frequency, code rate
	AGC values (if supported)	❖ AGC values
		❖ The output of the tracking stage (i.e., correlators value)
		❖ PSD estimation
		❖ Correlation distribution of the Chi-square GoF test)
		❖ IF digitalized samples
Capability	Detecting and Localizing the interference	❖ Detecting and Localizing the interference
		❖ Characterizing the interference
		❖ Replicating and simulating the real interference event by using transceiver front-end or GNSS simulator in replay mode
		❖ Analyzing the impact of interference in different stage of the GNSS receiver
		❖ Integrity monitoring [52,53]
Complexity	❖ Low complexity with the smartphone running application	❖ Require addition accessory such as GNSS front-end and antenna
	❖ The message sent to server is 929 bytes long	❖ The message sent to server is 9256 bytes long + IF digitalized samples (if interference detected) (*)

(*): The high-speed data transmission requirement can be satisfied in 4G/5G networks.

**Table 3:** Wideband interference: in-lab test results.

**WB Jammer Power**	−60 dBm	−80 dBm	−85 dBm	−95 dBm	−105 dBm
**TE (Total Energy of Error) (dB/Hz)^2^**	235,828	163,683	119,806	13,612	8409
**Receiver Signal Processing**	✕ Disrupted	✕ Severely compromised	✓ Slightly affected	✓ Unaffected	✓ Unaffected
**TE Threshold (dB/Hz)^2^**	50,000				
**Interference Detection**	YES	YES	YES	NO	NO

**Table 4:** Estimated false alarm rate of each signal vs. each reference distribution.

PRN under Test	C/N_0_ (dBHz)	PRN of the Reference Distribution Function							
		PRN 1 (−110 dBm)	PRN5 (−113 dBm)	PRN 6 (−116 dBm)	PRN 10 (−119 dBm)	PRN 16 (−122 dBm)	PRN 17 (−125 dBm)	PRN 21 (−128 dBm)	PRN22 (−131 dBm)
**PRN 1**	59	0	0	0.0025	0.0045	0.0134	0.2165	0.6914	0.7371
**PRN5**	55	0	0	0.0025	0	0	0.0135	0.5073	0.7371
**PRN 6**	52	0	0	0	0	0	0	0.2783	0.7371
**PRN 10**	48	0	0	0.0025	0	0	0	0.0449	0.7236
**PRN 16**	45	0.0179	0.0045	0.0270	0	0	0	0.0269	0.7101
**PRN 17**	42	0.5926	0.1389	0.0990	0.0492	0.0089	0	0	0.3056
**PRN 21**	39	0.7354	0.6764	0.3645	0.2950	0.0984	0.0045	0	0.0135
**PRN 22**	36	0.7354	0.7346	0.7368	0.7330	0.7159	0.4859	0.0045	0

**Table 5:** GoF test method: assignment of the pre-computed GPS L1 C/A reference distribution functions to C/N_0_ ranges of tracked signals.

Estimated C/N_0_ of the GPS L1 C/A Signal in Tracking (dBHz)	Assigned Reference Distribution (Associated C/N_0_ in dBHz)
>50	PRN 5 (55)
43–50	PRN 16 (45)
40–43	PRN 17 (42)
38–40	PRN 21 (39)
<38	PRN 22 (36)
